# Neuronal activity patterns in the mediodorsal thalamus and related cognitive circuits are modulated by metabotropic glutamate receptors

**DOI:** 10.1016/j.neuropharm.2014.12.031

**Published:** 2015-05

**Authors:** C.S. Copeland, S.A. Neale, T.E. Salt

**Affiliations:** a Institute of Ophthalmology, University College London, 11-43 Bath Street, London, EC1V 9EL, UK; b Neurexpert Ltd, Kemp House, City Road, London, EC1V 2NX, UK

**Keywords:** Mediodorsal thalamus, Metabotropic glutamate receptor, Synaptic inhibition, Burst firing, Schizophrenia, DMSO, dimethyl sulfoxide, GABA, gamma-amino butyric acid, i.p., intraperitoneal, LY354740, (1S,2S,5R,6S)-2-Aminobicyclo[3.1.0]hexane-2,6-dicarboxylic acid, LY487379, 2,2,2-Trifluoro-N-[4-(2-methoxyphenoxy)phenyl]-N-(3-pyridinylmethyl)ethanesulfonamide hydrochloride, MD, mediodorsal thalamus, mGlu, metabotropic glutamate, mGlu2, metabotropic glutamate subtype 2, mGlu3, metabotropic glutamate subtype 3, NaCl, sodium chloride, NMDA, N-methyl d-aspartate, PAM, positive allosteric modulator, PFC, prefrontal cortex, PSTH, post-stimulus time histogram, TRN, thalamic reticular nucleus, VB, ventrobasal thalamus

## Abstract

The mediodorsal thalamus (MD) likely plays an important role in cognition as it receives abundant afferent connections from the amygdala and prefrontal cortex (PFC). Indeed, disturbed activity within the MD is thought to precipitate cognitive deficits associated with schizophrenia. As compounds acting at the Group II metabotropic glutamate (mGlu) receptors (subtypes mGlu2/mGlu3) have efficacy in animal models of schizophrenia, we investigated whether a Group II agonist and an mGlu2 positive allosteric modulator (PAM) could modulate MD activity. Extracellular single-unit recordings were made *in vivo* from MD neurones in anaesthetised rats. Responses were elicited by electrical stimulation of the PFC and/or amygdala, with Group II compounds locally applied as required. The Group II agonist reduced inhibition evoked in the MD: an effect manifested as an increase in short-latency responses, and a decrease in long-latency burst-firing. This disinhibitory action of the Group II receptors in the MD represents a mechanism of potential therapeutic importance as increased inhibition in the MD has been associated with cognitive deficit-onset. Furthermore, as co-application of the mGlu2 PAM did not potentiate the Group II agonist effects in the MD, we suggest that the Group II disinhibitory effect is majority-mediated via mGlu3. This heterogeneity in Group II receptor thalamic physiology bears consequence, as compounds active exclusively at the mGlu2 subtype are unlikely to perturb maladapted MD firing patterns associated with cognitive deficits, with activity at mGlu3 receptors possibly more appropriate. Indeed, polymorphisms in the mGlu3, but not the mGlu2, gene have been detected in patients with schizophrenia.

## Highlights

• There is heterogeneity in Group II receptor physiology across thalamic nuclei.
• This differential distribution may facilitate multimodal thalamic nuclei functions.
• Group II receptor activation reduced burst firing via reducing thalamic inhibition.
• Increased thalamic inhibition precipitates impairments in cognitive function.
• Activating the Group II receptors may therefore enhance cognitive function.

## 1 Introduction

Schizophrenia is a devastating psychiatric disorder with a population prevalence of 1%. Whilst the core clinical symptoms of psychosis present as ‘positive’ symptoms, those in addition to normal behaviuor, (hallucinations, paranoia, delusions) or ‘negative’ symptoms, the absence of it, (depression, social withdrawal, anhedonia), schizophrenia is also closely associated with deficits in a wide range of cognitive processes, including working memory, executive function and attention (Kay et al., 1987; Lewis and Lieberman, 2000). It has been postulated that abnormal activity in specific brain regions, such as the cortex, hippocampus and thalamus (Review: Barch and Ceaser (2012)), and/or neurotransmitter systems, including glutamatergic and serotinergic signalling pathways (Moghaddam and Adams, 1998; Aghajanian and Marek, 1999), are associated with these distinct symptoms and signs. Current therapies fail to alleviate cognitive impairments, even though they constitute the major determinants for the psychosocial functioning of patients with the disorder (Zipursky, 2014). Identifying the aetiology underlying cognitive dysfunction is therefore a prerequisite for the development of future treatments for patients with schizophrenia.

Thalamocortical synchronization is thought to play a crucial role in the gating and processing of sensory, motor and cognitive information (Saalmann et al., 2012). Anatomical connections made between the thalamus and cortex are thought to be key in the regulation of this thalamocortical synchronization: all thalamic nuclei project thalamocortical afferents to layer IV of the cortex, and also receive reciprocal corticothalamic inputs from layer VI, which modulate how information is transmitted by the thalamus (Guillery, 1995; Sherman and Guillery, 2001). In addition, both thalamocortical and corticothalamic afferents innervate the associated thalamic reticular nucleus (TRN), which projects to and provides a GABAergic inhibition in the relevant thalamic nucleus (Guillery, 1995; Sherman and Guillery, 2001). The mediodorsal thalamus (MD) is considered crucial in the regulation of key aspects of cognition due to the afferents it receives from the amygdala and abundant modulatory connections it receives from the prefrontal cortex (PFC) (Kuroda et al., 1998; Jones, 2007). As both anatomical and functional abnormalities have been consistently detected in the thalamus of patients with schizophrenia (Cronenwett and Csernansky, 2010), disturbed activity within the MD has been identified as a key circuitry component postulated to underlie neuropathological alterations that precipitate the characteristic cognitive abnormalities of the disease (Andrews et al., 2006; Mitelman et al., 2006; Minzenberg et al., 2009; Marenco, 2012; Woodward et al., 2012; Parnaudeau et al., 2013).

The metabotropic glutamate (mGlu) receptors enable the major excitatory neurotransmitter, glutamate, to play a regulatory role in neural communication. Specifically, the Group II mGlu receptors (mGlu2 and mGlu3) have been identified as novel targets in the treatment of schizophrenia upon the inaugural discovery that administration of the Group II selective orthosteric agonist LY354740 ameliorates psychotic behaviours (Moghaddam and Adams, 1998; Aghajanian and Marek, 1999). Since then, several preclinical studies using selective Group II compounds (see Review: Herman et al., 2012), have established the Group II mGlu receptors as a novel potential target for a new class of antipsychotic drug. Furthermore, Group II mGlu receptors are moderately/highly expressed in limbic brain regions in healthy controls, including the MD (Petralia et al., 1996; Wright et al., 2001; Gu et al., 2008).

In the present study, where we performed a series of *in vivo* recording experiments, we sought to investigate how activation of the Group II mGlu receptors affects thalamic activity in the MD upon stimulation of the amygdala and/or PFC. In addition, as the Group II mGlu receptors have been previously demonstrated to modulate thalamic responses to somatosensory input (Salt and Turner, 1998; Copeland et al., 2012), we also conducted complementary comparative experiments in the somatosensory ventrobasal thalamus (VB). The findings of this study were two-fold: firstly, the data suggest that the Group II mGlu receptors function within the MD to disinhibit thalamic neurones: a mechanism of potential therapeutic importance as increased inhibition in the MD has been associated with cognitive deficit-onset (Parnaudeau et al., 2013); and secondly, that Group II mGlu receptor distribution across thalamic nuclei is not uniform. Taken together these data can lead us to suggest that compounds active exclusively at the mGlu2 receptor are unlikely to perturb any maladapted MD firing patterns associated with cognitive deficits, with activity at mGlu3 receptors likely more appropriate.

## 2 Materials and methods

### 2.1 Animals & experimental procedures

All experiments were conducted using adult male Wistar rats (290–450 g, n = 13). Animals (Harlan, UK) were housed on a 12 h light/dark cycle with food and water *ad libitum*. All experimental conditions and procedures were approved by the Home Office (UK) and were in accordance with the UK Animals (Scientific Procedures) Act 1986 and associated guidelines. All studies complied with the ARRIVE guidelines. All efforts were made to minimize animal suffering and to reduce the number of animals used.

### 2.2 Surgery

Animals were anaesthetised with urethane (1.2 g/kg intraperitoneal [i.p.] injection) and were prepared for recording as previously described (Salt, 1989). Throughout the experiments, electroencephalogram and electrocardiogram were monitored. Additional urethane anaesthetic was administered i.p. as required, and the experiment was terminated with an overdose of the same anaesthetic.

### 2.3 Recording and iontophoresis

Seven-barrel recording and iontophoretic glass pipettes were advanced into the MD or VB. Extracellular recordings were made from single MD or VB neurones through the central barrel (filled with 4 M sodium chloride [NaCl]). Iontophoretic drug applications were performed using the outer barrels (Salt, 1989). On each occasion, one of the outer barrels was filled with 1 M NaCl for current balancing. The remaining outer barrels each contained one of the following substances: NMDA (50 mM, pH 8.0 in 150 mM NaCl); (1S,2S,5R,6S)-2-Aminobicyclo[3.1.0]hexane-2,6-dicarboxylic acid (LY354740; 5 mM, pH 8.0 in 75 mM NaCl) and pontamine sky blue dye (2% in 1 M NaCl) as Na^+^ salts ejected as anions, with 2,2,2-Trifluoro-N-[4-(2-methoxyphenoxy)phenyl]-N-(3-pyridinylmethyl)ethanesulfonamide hydrochloride (LY487379; 1 mM, pH 6.0, in 1% dimethyl sulfoxide [DMSO], 75 mM NaCl) ejected as cations. All compounds were prevented from diffusing out of the pipette by using a retaining current (10–20 nA) of opposite polarity to that of the ejection current. Compounds were ejected within a current range ensured to produce a sub-maximal effect on thalamic inhibition (LY354740 6–50 nA; LY487379 50–100 nA). Pontamine sky blue was ejected at the end of each MD experiment to enable identification of the recording site location. All compounds were obtained from Tocris (Bristol, UK).

### 2.4 Stimulation protocols

#### 2.4.1 Electrical stimulation

Neurones were identified as MD neurones for experimental purposes on the basis of stereotaxic location (AP +4.0 mm from lambda; ML 0.5 mm) (Paxinos and Watson, 1998) and responses to electrical stimulation of the PFC and/or amygdala. All electrode sites were confirmed histologically, with stimulation sites identified by electrode tracks, and recording sites identified by pontamine sky blue dye spots. Data collected from incorrect electrode placements were discarded. Electrical stimulation of the PFC was performed using insulated tungsten wires located 0.8–1 mm apart (0.2 ms, 1–10 V, square pulses), whilst electrical stimulation of the amygdala was performed using bipolar concentric electrodes (0.2 ms, 1–10 V, square pulses). Both types of electrode were advanced into their appropriate stereotaxic location using micromanipulators (PFC: AP +9.5 mm from lambda; ML 0.8 mm; amygdala AP +1.7 mm from lambda; ML 3.7 mm at a 21° angle) (Paxinos and Watson, 1998). Using such an approach it is possible to use electrical stimulation of the PFC and amygdala to evoke either excitatory or inhibitory responses, as described previously (Fernandez de Molina and Ispizua, 1972; Sidorov and Podachin, 1982). Recordings were made from both quiescent and spontaneously firing neurones, with the experimental protocol performed adjusted accordingly.

Cycles of electrical stimulation (10 s long) were established and repeated continuously whilst recording from MD neurones. Cycles consisted of alternating electrical stimulation of the PFC and amygdala with a 4–5 s interstimulus interval. After several control cycles displaying consistent neurone responses had been recorded, LY354740 and LY487379 were iontophoretically ejected either alone or in conjunction with each other for 2–14 min using parameters that we have previously found to be effective (Copeland et al., 2012). After cessation of LY354740 and/or LY487379 ejection, electrical stimulation cycles were continued until neurone responses had returned to control levels. An inter-stimulus interval of 4–5 s was sufficient to ensure that any post-stimulus effects from either stimulus type were no longer apparent upon subsequent stimulation (Salt, 1989; Turner and Salt, 2003).

#### 2.4.2 Vibrissa deflection

Neurones were identified as VB neurones on the basis of stereotaxic location (Paxinos and Watson, 1998) and responses to vibrissa deflection. Vibrissa deflection was performed using fine air jets directed through 23 gauge needles mounted on micromanipulators positioned and orientated close to the vibrissa to ensure deflection of a single vibrissa was achieved. Air jets were electronically gated with solenoid valves that produced a rising air pulse at the vibrissa 8 ms after switching. Response latencies were calculated from the start of the gating pulse. Using such an approach it is possible to use air jets on adjacent vibrissae and only evoke an excitatory response from one of the vibrissa stimuli, indicating the specificity of the stimulation procedure, as described previously (Salt, 1989). Prior to the beginning of each experimental protocol, the ‘principal’ vibrissa (i.e. the vibrissa at the centre of the receptive field) for each neurone was identified, and responses to additional vibrissae were noted. All VB neurones recorded from were quiescent.

Cycles of sensory stimulation (10 s long) were established and repeated continuously whilst recording from neurones. Cycles consisted of electronically gated short (10–30 ms) duration air jets directed at the principal vibrissa, with a 4–5 s interstimulus interval. After several control cycles displaying consistent neurone responses had been recorded, LY354740 and LY487379 were iontophoretically ejected either alone or in conjunction with each other for 2–12 min as required. After cessation of LY354740 and/or LY487379 ejection, sensory stimulation cycles were continued until neurone responses had returned to control levels. An inter-stimulus interval of 4–5 s was sufficient to ensure that any post-stimulus effects from either stimulus type were no longer apparent upon subsequent stimulation (Salt, 1989; Turner and Salt, 2003).

### 2.5 Data collection and statistical analysis

Throughout the study, extracellular single neurone action potentials were gated, timed and counted using a window discriminator, a CED1401 interface and Spike2 software (Cambridge Electronic Design, Cambridge, UK), which recorded the output from the iontophoresis unit and also triggered the iontophoretic, electrical and vibrissa deflection stimulation sequences. Data were analysed by plotting post-stimulus histograms (PSTHs) from these recordings by counting the spikes evoked upon either electrical stimulation or vibrissa deflection. We used conventional criteria to divide neuronal responses into burst and tonic activity (Lu et al., 1992; Wang et al., 2006). These required that before the first action potential in a burst, there was a preceding silent period of at least 100 ms, which was then followed by a second spike with an interspike interval ≤4 ms. Any subsequent action potentials with preceding interspike intervals ≤4 ms were also considered to be part of a burst. All other spikes were regarded as tonic. We computed a burst-tonic firing ratio (the proportion of burst spikes normalized with respect to the total number of recorded spikes). Data are expressed as a percentage of control responses prior to compound application (±SEM). Comparisons were made using Wilcoxon matched-pairs test (*p* < 0.01).

## 3 Results

### 3.1 Neuronal population

Data were collected from 19 neurones located in the MD that were responsive to electrical stimulation of the PFC and/or amygdala, and 5 neurones in the VB that were responsive to vibrissa defection in urethane-anaesthetised rats. It has previously demonstrated that urethane has little effect on observed neuronal responses when compared to recordings taken from neurons in unanaesthetised rats (Holmes and Houchin, 1966; Simon et al., 2006). Due to the absence of interneurones in the rodent MD (Kuroda et al., 1998) and VB (Ralston, 1983; Barbaresi et al., 1986; Harris and Hendrickson, 1987; Ohara and Lieberman, 1993), all recordings can be presumed to be from thalamocortical neurones. Both quiescent (68%) and spontaneously active (32%) MD neurones were recorded from, which is consistent with MD neurone population activity previously reported (Fernandez de Molina and Ispizua, 1972; Sidorov and Podachin, 1982). The magnitude of evoked responses (in terms of evoked action potentials) in the MD upon stimulation of the PFC was, on average, much greater than that evoked upon stimulation of the amygdala. This may arise from the abundant projections from the PFC innervating the MD to a larger degree than the sparser projections from the amygdala (Kuroda et al., 1998; Jones, 2007). Single short-latency (6–50 ms) spikes were evoked in the MD upon stimulation of both the PFC and amygdala, with long latency (300–200 ms) bursts also evoked in the MD upon stimulation of the PFC only: response parameters consistent with those previously observed. The distribution of the evoked response latencies into these two broad ranges is similar to the previously reported MD population activity (Fernandez de Molina and Ispizua, 1972; Sidorov and Podachin, 1982). In the VB, recordings were made from quiescent neurones in which long-latency (300–400 ms) bursts comprising 2–6 spikes were evoked upon vibrissal deflection. Responses evoked in quiescent VB neurones upon short-duration vibrissa deflection, which have been previously reported (Copeland et al., 2012), are also referred to.

### 3.2 Group II mGlu receptors can reduce inhibition evoked in the MD

**Fig. 1:**
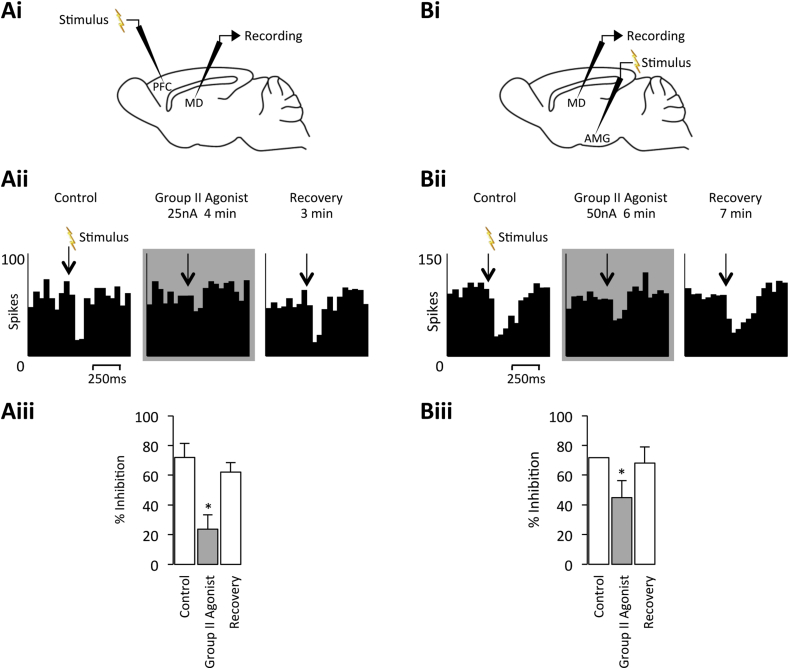
**Group II mGlu receptor activation reduces inhibition evoked in the MD**. **Ai** Stimulation and recording sites for the PFC and MD electrodes, respectively. **Aii** Peristimulus time histograms (PSTHs) of responses of a spontaneously firing MD neurone (CMD31a) to electrical stimulation of the PFC under normal conditions, upon Group II agonist application, and recovery. 50 ms bins over 30 trials. **Aiii** Bars represent the mean % response (±SEM) under the same conditions (n = 5). *p < 0.05 when compared to control. **Bi** Stimulation and recording sites for the amygdala and MD electrodes, respectively. **Bii** PSTHs of responses of a spontaneously firing MD neurone (CMD02b) to electrical stimulation of the amygdala under normal conditions, upon Group II agonist application, and recovery. 50 ms bins over 30 trials. **Biii** Bars represent the mean % response (±SEM) under the same conditions (n = 6). *p < 0.05.

We first assessed whether the Group II mGlu receptors could broadly disinhibit neuronal firing in the MD. Conveniently, spontaneously-firing MD neurones provided us with a background of excitation upon which inhibition could be visualised. Stimulation of either the PFC or amygdala was found to reduce the spontaneous firing of MD neurones, with local application of the Group II mGlu receptor orthosteric agonist LY354740 able to significantly reduce the extent of the evoked inhibition (PFC – 24% ± 10% of control; n = 6 from 5 rats; *p* < 0.05; amygdala – 45% ± 12% of control; n = 5 from 5 rats; *p* < 0.05; Fig. 1). The ability of the Group II mGlu receptors to reduce evoked inhibition in a thalamic nucleus is similar to that observed previously in the VB (Salt and Turner, 1998; Copeland et al., 2012). Therefore, we next sought to examine how Group II mGlu receptor activation may modulate characteristic thalamic activity patterns, short-latency and long-latency burst firing, in the MD.

### 3.3 Group II mGlu receptor modulation of evoked short-latency and long-latency burst firing patterns of MD neurones

It is well known that the firing pattern of thalamic neurones exhibits two distinct response patterns: short-latency and long-latency burst firing (although both firing patterns can be seen together in varying proportions (Ramcharan et al., 2000; Rivadulla et al., 2003). Short-latency responses are associated with a linear transmission of information, and occurs when thalamic neurones have been depolarised from resting potential, and follows the inactivation of a voltage- and time-dependent calcium current (*I*_T_), whilst long-latency burst-mode firing occurs when there has been a sustained hyperpolarisation of thalamic neurones for 100 ms or more and *I*_T_ is de-inactivated (Llinas and Jahnsen, 1982; Jahnsen and Llinas, 1984). The effect of Group II mGlu receptor activation on both short-latency and long-latency burst firing patterns was therefore assessed.

**Fig. 2:**
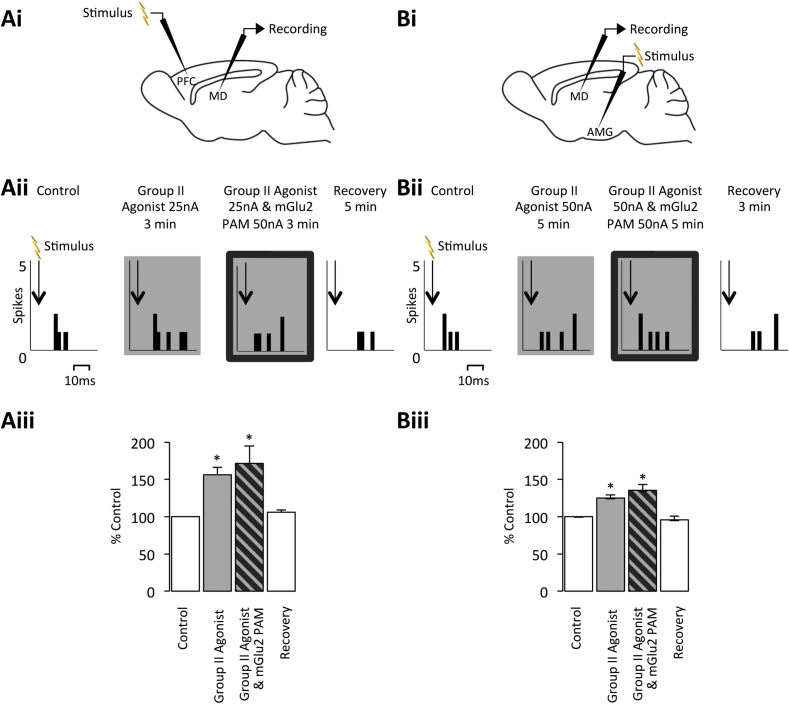
**Group II mGlu receptor activation increases evoked tonic firing in the MD**. **Ai** Stimulation and recording sites for the PFC and MD electrodes, respectively. **Aii** PSTHs of responses of a tonically firing MD neurone (CMD22a) to electrical stimulation of the PFC under normal conditions, upon Group II agonist application alone, upon Group II agonist and mGlu2 PAM co-application, and recovery. 2 ms bins over 18 trials. **Aiii** Bars represent the mean % response (±SEM) under the same conditions (n = 5). *p < 0.05 when compared to control. **Bi** Stimulation and recording sites for the amygdala and MD electrodes, respectively. **Bii** PSTHs of responses of a tonically firing MD neurone (CMD34a) to electrical stimulation of the amygdala under normal conditions, upon Group II agonist application alone, upon Group II agonist and mGlu2 PAM co-application, and recovery. 2 ms bins over 18 trials. **Biii** Bars represent the mean % response (±SEM) under the same conditions (n = 5). *p < 0.05.

In quiescent MD neurones in which short-latency firing could be evoked, local application of the Group II agonist was able to significantly increase short-latency neuronal responses upon electrical stimulation of either the PFC or amygdala (PFC – 152% ± 8% of control; n = 5 from 4 rats; *p* < 0.05; amygdala – 124% ± 6% of control; n = 5 from 3 rats; *p* < 0.05; Fig. 2). This increase in excitatory response is likely due to Group II mGlu receptors localized on TRN terminals reducing GABAergic transmission and subsequent thalamic inhibition (Ohara and Lieberman, 1993; Varga et al., 2002). However, in the same population of neurones, co-application of the mGlu2 PAM did not potentiate the Group II agonist effect on the evoked response to either stimulation (PFC – 171% ± 24% of control; n = 5 from 4 rats; *p* > 0.05; amygdala – 135% ± 8% of control; n = 5 from 3 rats; *p* > 0.05; Fig. 2), indicating that there is no mGlu2 component to the overall Group II mGlu receptor effect in the MD. This is in contrast to the Group II mGlu receptor modulation of physiologically-evoked short-latency activity in the VB, which has been demonstrated to comprise an mGlu2 receptor component (Copeland et al., 2012).

**Fig. 3:**
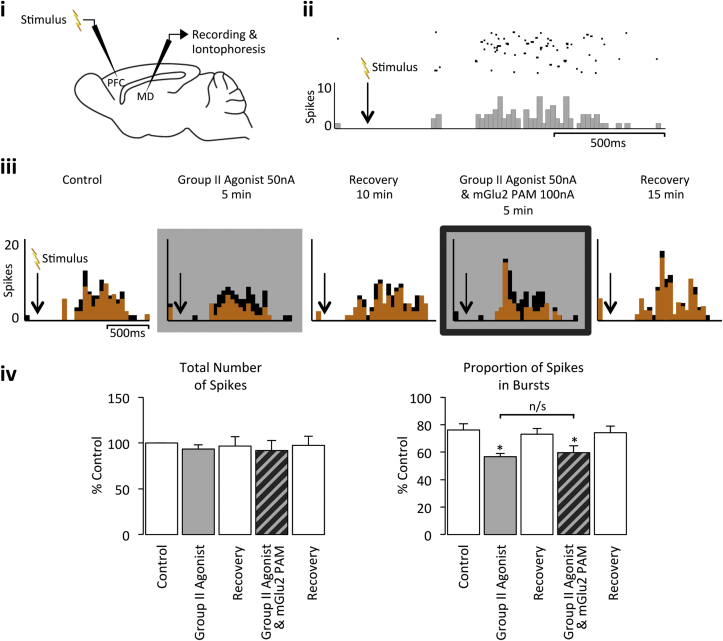
**Group II mGlu receptor activation decreases evoked burst firing in the MD**. **i** Stimulation and recording sites for the PFC and MD electrodes, respectively. **ii** Illustrative raster display and PSTH of an MD neuron (CMD16a) burst firing in response to PFC stimulation. 20 ms bins over 30 trials. **iii** PSTHs of burst firing responses of the same MD neurone to electrical stimulation of the PFC under normal conditions, upon Group II agonist application alone, upon Group II agonist and mGlu2 PAM co-application, and recovery. Burst spikes, orange; non-burst spikes, black; 50 ms bins over 30 trials. **iv** Bars represent the mean % response (±SEM) under the same conditions (n = 5). *p < 0.05 when compared to control; n/s, not significant. (For interpretation of the references to colour in this figure legend, the reader is referred to the web version of this article.)

**Fig. 4:**
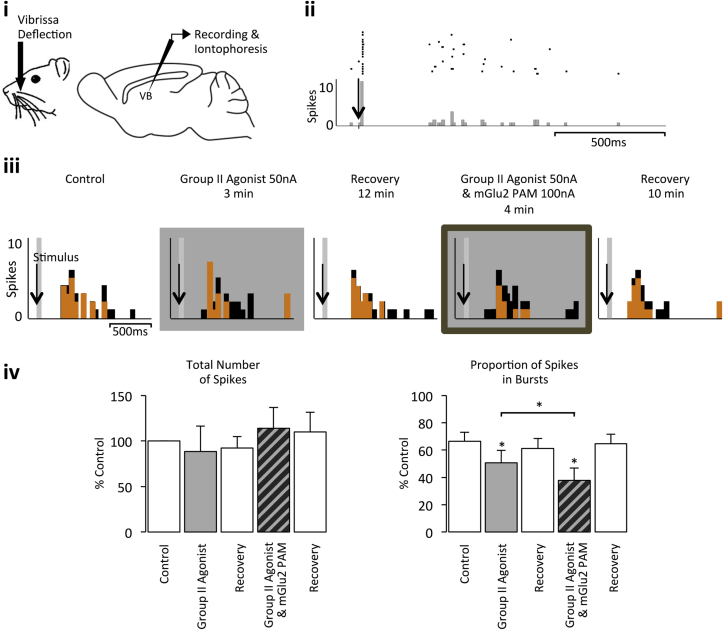
**Group II mGlu receptor activation decreases evoked burst firing in the VB**. **i** Vibrissal deflection schematic and recording site location for the VB electrode. **ii** Illustrative raster display and PSTH of a VB neuron (CVB78c) displaying both tonic and burst firing in response to vibrissal deflection. 20 ms bins over 30 trials. **iii** PSTHs of burst firing responses of the same VB neurone to vibrissal deflection under normal conditions, upon Group II agonist application alone, upon Group II agonist and mGlu2 PAM co-application, and recovery. Burst spikes, orange; tonic short-latency (8–50 ms) spikes, grey; total long-latency (300–1000 ms) spikes, black; 50 ms bins over 30 trials. **iv** Bars represent the mean % response (±SEM) under the same conditions (n = 5). *p < 0.05 when compared to control, unless otherwise indicated. (For interpretation of the references to colour in this figure legend, the reader is referred to the web version of this article.)

In quiescent MD neurones in which long-latency burst-firing could be evoked, local application of the Group II agonist was also able to significantly reduce the proportion of burst activity evoked upon electrical stimulation of the PFC without affecting the overall magnitude of the response (Control – total number of spikes: 100% ± 0%; proportion of spikes in bursts: 76% ± 5%; Group II agonist – total number of spikes: 93% ± 5%; proportion of spikes in bursts: 57% ± 2%; n = 5 from 4 rats, *p* < 0.05; Fig. 3). This decrease in the proportion of burst firing is likely due to Group II mGlu receptors localized on TRN terminals reducing GABAergic transmission and the subsequent hyperpolarization of MD neurones (Llinas and Jahnsen, 1982; Jahnsen and Llinas, 1984; Ohara and Lieberman, 1993; Varga et al., 2002). However, in the same population of neurones, co-application of the mGlu2 PAM did not potentiate the Group II agonist effect on evoked burst activity (Group II agonist plus mGlu2 PAM – total number of spikes: 92% ± 11% of control; proportion of spikes in bursts: 60% ± 5%; n = 5 from 4 rats; *p* > 0.05; Fig. 3). In contrast, in quiescent VB neurones in which burst-firing could be evoked, local application of the Group II agonist was able to significantly reduce burst activity evoked upon principal vibrissa deflection without affecting the magnitude of the overall neuronal response (Control – total number of spikes: 100% ± 0%; proportion of spikes in bursts 66% ± 7%; Group II agonist – total number of spikes: 88% ± 28%; proportion of spikes in bursts 51% ± 9%; n = 5 from 5 rats, *p* < 0.05; Fig. 4); an effect that was potentiated upon co-application of the mGlu2 PAM (Group II agonist plus mGlu2 PAM – total number of spikes: 114% ± 23% of control; proportion of spikes in bursts 38% ± 9%, n = 5 from 5 rats, *p* < 0.05; Fig. 4). These data provide further evidence that whilst there is an mGlu2 component to the Group II mGlu receptor effect in the VB, there is no such component in the MD.

## 4 Discussion

Group II mGlu receptor function in the somatosensory rodent thalamus has been investigated extensively (Salt and Eaton, 1995a; b; Salt et al., 1996; Salt and Turner, 1998; Turner and Salt, 2003; Copeland et al., 2012). However, whether this function represents an over-arching principle of thalamic physiology is not known. The data obtained in this study using *in vivo* electrophysiology and iontophoresis clearly demonstrate that Group II mGlu receptor activity disinhibits neuronal responses in the rat MD, and that there is heterogeneity in Group II mGlu receptor physiology across thalamic nuclei. As increased inhibition in the MD has been associated with cognitive deficit-onset (Parnaudeau et al., 2013), these results may influence the design of future Group II mGlu receptor therapies as compounds active exclusively at the mGlu2 subtype are unlikely to perturb maladapted MD firing patterns associated with cognitive deficits, with activity at mGlu3 receptors likely more appropriate.

LY354740 is the best-studied selective Group II mGlu receptor orthosteric agonist (Schoepp et al., 2003), and has been extensively used to probe Group II mGlu receptor function in behavioural (Schoepp et al., 2003; Nordquist et al., 2008) and physiological (Flor et al., 2002; Moldrich et al., 2003) studies in both the human and rodent CNS *in vivo* and *in vitro*. LY487379, a highly selective mGlu2 PAM, which possesses no intrinsic agonist activity but does enhance responses to submaximal glutamate without activity at other receptors or ion channels (Johnson et al. 2003), has been used in behavioural and *in vitro* electrophysiological studies in the rodent CNS (Schaffhauser et al., 2003; Galici et al., 2005; Poisik et al., 2005; Harich et al., 2007; Hermes and Renaud, 2010; Nikiforuk et al., 2010). LY487379 possesses higher selectivity for the mGlu2 receptor than the orthosteric Group II mGlu receptor antagonist LY341495 (Kingston et al., 1998; Schoepp et al., 1999), making LY487379 the most appropriate selective mGlu2 receptor compound to be used in conjunction with LY354740 in this study. Furthermore, given our previous findings with LY487379 in the somatosensory thalamus (Copeland et al., 2012), it was appropriate to carry out similar studies in the MD with this agent. The pharmacological specificity of our drug applications is clearly crucial to the interpretation of the results of the present study. The iontophoretic parameters used for LY354740 and LY487379 in this study have been demonstrated by ourselves to apply pharmacologically appropriate quantities (Copeland et al., 2012). Furthermore, application of either LY354740 or LY487379 has been demonstrated to have no effect on responses evoked by NMDA or AMPA in thalamic neurones, indicating that non-specific effects are not being produced by our drug application protocols (Copeland et al., 2012).

### 4.1 Group II mGlu receptor function across thalamic nuclei is not uniform

It is well established that the Group II mGlu receptors can modulate somatosensory transmission in the rat VB by reducing inhibitory drive from the associated TRN (Salt and Eaton, 1995a, b; Salt et al., 1996; Salt and Turner, 1998; Turner and Salt, 2003; Copeland et al., 2012); a mechanism that comprises an mGlu2 receptor component (Copeland et al., 2012). It has been postulated that these mGlu2 receptors function in a highly specific manner to enable relevant sensory information to be discerned from background activity (Copeland et al., 2012): a mechanism whose malfunctioning could result in maladaption of sensory perception, such as that which can occur in psychiatric disease, such as schizophrenia (Javitt, 2009; Saalmann and Kastner, 2011). This novel mechanism could therefore also be of potential importance in attentional and cognitive processes in other thalamic nuclei. However, the data presented in this study suggest that whilst there is a disinhibition of MD neurone responses to PFC and amygdala afferent stimulation upon Group II mGlu receptor activation, this modulation does not comprise an mGlu2 component. This heterogeneity in Group II mGlu receptor physiology may represent a key component in the facilitation of the multimodal functions possessed by different thalamic nuclei. The primary method used by rodents to explore their surroundings is via feedback from vibrissal deflections (see Review: Diamond et al., 2008). It is therefore of paramount importance that sensory discrimination between vibrissal deflections is enhanced to enable optimal object localization: a mechanism that is likely facilitated by mGlu2 receptor activation. In comparison to the VB, which only receives afferent inputs from the vibrissal system, the MD receives projections originating from a large number of cortical and subcortical structures including the PFC, the amygdala, the nucleus of the diagonal band of Broca, the ventral pallidum, the dorsolateral tegmental nucleus and the pars reticulata of the substantia nigra (see Review: (Kuroda et al., 1998)). As the MD is thought to function as an integrator of these afferent inputs before transmitting them as coherent information to the PFC (Watanabe and Funahashi, 2012; Uhlhaas et al., 2013), a scenario in which mGlu2 receptor activation would enhance inputs from one brain area whilst reducing those from another is unlikely to optimise the integrative function of the MD.

### 4.2 mGlu3 receptor activation can modulate MD neuronal firing patterns

The two different firing patterns of thalamic neurones – short-latency (tonic) responses and long-latency burst firing – are associated with distinct patterns of information transfer from thalamus to cortex (Fernandez de Molina and Ispizua, 1972; Llinas and Jahnsen, 1982; Jahnsen and Llinas, 1984; Ramcharan et al., 2000; Rivadulla et al., 2003). Short-latency responses predominate when thalamic neurons have been depolarized from resting potential, with impulses occurring at high and regular rates, and where synaptic transmission through the thalamus is faithfully relayed. In contrast, transmission through the thalamus is less reliable upon long-latency burst firing, which predominates when thalamic neurones have been hyperpolarized for 100 ms or more, with impulses occurring at low and irregular rates punctuated by high-frequency bursts. It has been recently demonstrated that a subtle hyperpolarisation of MD neurones is sufficient to trigger selective impairments in prefrontal-dependent cognitive behaviours in rodents (Parnaudeau et al., 2013). In the present study, Group II mGlu receptor activation in both the MD and VB was able to significantly reduce burst firing upon either vibrissa deflection or PFC and/or amygdala stimulation, respectively. In both nuclei, this is likely due to Group II mGlu receptors localized on TRN terminals reducing GABAergic transmission and subsequent thalamic inhibition (Ohara and Lieberman, 1993; Varga et al., 2002). Indeed, we were able to demonstrate that the amount of inhibition evoked in the MD upon PFC and/or amygdala stimulation was significantly reduced by Group II agonist application; an effect which has also been observed in the VB (Copeland et al., 2012). As burst firing is associated with a sustained hyperpolarisation of thalamic neurones, a reduction in inhibitory drive would be expected to decrease burst-firing activity. Several preclinical studies (see Review: Herman et al., 2012), have indicated that selective targeting of the Group II mGlu receptors may represent a novel target to treat some of the symptoms associated with schizophrenia. Therefore, it is appropriate to postulate that their mechanism of action may be to reduce burst firing within thalamic nuclei via a reduction in inhibitory drive from the TRN to ensure synchronous activity between the cortex and thalamus. Indeed, functional magnetic resonance imaging studies have consistently detected altered correlation between activity in the MD and PFC at rest and during cognitive tasks (Mitelman et al., 2005; Minzenberg et al., 2009; Woodward et al., 2012). These studies suggest that altered MD activity and/or impaired communication between the MD and PFC could play a role in the cognitive deficits seen in patients with schizophrenia. However, whilst the Group II mGlu receptors are moderately/highly expressed in limbic brain regions in healthy controls, (Petralia et al., 1996; Wright et al., 2001; Gu et al., 2008), the data presented here indicate that targeting the mGlu3 receptor would be advantageous, as no mGlu2 activity was detected in the MD. Indeed, the mGlu3 receptor has been implicated in the aetiological, pathophysiological and pharmacotherapeutic aspects of the disorder (Harrison et al., 2008), with polymorphisms in the mGlu3 receptor gene and protein, but not the mGlu2 receptor, detected in patients with schizophrenia (Ghose et al., 2009; Cherlyn et al., 2010; Mounce et al., 2014). Several clinical trials have been conducted to assess the efficacy of agonists targeting both mGlu2 and mGlu3 receptor subtypes to treat schizophrenia symptoms (Patil et al., 2007; Kinon et al., 2011; Adams et al., 2013; Stauffer et al., 2013), with varying success. Taking into account the results presented here, the design of future novel therapies targeted to treat deficits in cognitive function may therefore achieve greater success if selectivity and higher efficacy for mGlu3 receptors were achieved.

### 4.3 Conclusions

The significance of the results obtained in this study is two-fold: firstly, the data suggests that the Group II mGlu receptors function within the MD to disinhibit thalamic neurones: a mechanism of potential therapeutic importance as increased inhibition in the MD has been associated with cognitive deficit-onset; and secondly, that Group II mGlu receptor distribution across thalamic nuclei is not uniform. Taken together these data can lead us to suggest that compounds active exclusively at the mGlu2 receptor are unlikely to perturb any maladapted MD firing patterns associated with cognitive deficits, with activity at mGlu3 receptors likely more appropriate.

## Authorship contributions

Participated in research design: Copeland, Neale, and Salt
Conducted experiments: Copeland
Performed data analysis: Copeland
Wrote or contributed to the writing of the manuscript: Copeland, Neale, and Salt.

## References

[bib1] Adams D.H., Kinon B.J., Baygani S., Millen B.A., Velona I., Kollack-Walker S., Walling D.P. (2013). A long-term, phase 2, multicenter, randomized, open-label, comparative safety study of pomaglumetad methionil (LY2140023 monohydrate) versus atypical antipsychotic standard of care in patients with schizophrenia. BMC Psychiatry.

[bib2] Aghajanian G.K., Marek G.J. (1999). Serotonin, via 5-HT2A receptors, increases EPSCs in layer V pyramidal cells of prefrontal cortex by an asynchronous mode of glutamate release. Brain Res..

[bib3] Andrews J., Wang L., Csernansky J.G., Gado M.H., Barch D.M. (2006). Abnormalities of thalamic activation and cognition in schizophrenia. Am. J. Psychiatry.

[bib4] Barbaresi P., Spreafico R., Frassoni C., Rustioni A. (1986). GABAergic neurons are present in the dorsal column nuclei but not in the ventroposterior complex of rats. Brain Res..

[bib5] Barch D.M., Ceaser A. (2012). Cognition in schizophrenia: core psychological and neural mechanisms. Trends Cogn. Sci..

[bib6] Cherlyn S.Y., Woon P.S., Liu J.J., Ong W.Y., Tsai G.C., Sim K. (2010). Genetic association studies of glutamate, GABA and related genes in schizophrenia and bipolar disorder: a decade of advance. Neurosci. Biobehav. Rev..

[bib7] Copeland C.S., Neale S.A., Salt T.E. (2012). Positive allosteric modulation reveals a specific role for mGlu2 receptors in sensory processing in the thalamus. J. Physiol..

[bib8] Cronenwett W.J., Csernansky J. (2010). Thalamic pathology in schizophrenia. Curr. Top. Behav. Neurosci..

[bib9] Diamond M.E., von Heimendahl M., Knutsen P.M., Kleinfeld D., Ahissar E. (2008). 'Where' and 'what' in the whisker sensorimotor system. Nat. Rev. Neurosci..

[bib10] Fernandez de Molina A., Ispizua A. (1972). Neuronal activity evoked in nucleus medialis dorsalis of the thalamus after stimulation of the amygdala. Physiol. Behav..

[bib11] Flor P.J., Battaglia G., Nicoletti F., Gasparini F., Bruno V. (2002). Neuroprotective activity of metabotropic glutamate receptor ligands. Adv. Exp. Med. Biol..

[bib12] Galici R., Echemendia N.G., Rodriguez A.L., Conn P.J. (2005). A selective allosteric potentiator of metabotropic glutamate (mGlu) 2 receptors has effects similar to an orthosteric mGlu2/3 receptor agonist in mouse models predictive of antipsychotic activity. J. Pharmacol. Exp. Ther..

[bib13] Ghose S., Gleason K.A., Potts B.W., Lewis-Amezcua K., Tamminga C.A. (2009). Differential expression of metabotropic glutamate receptors 2 and 3 in schizophrenia: a mechanism for antipsychotic drug action?. Am. J. Psychiatry.

[bib14] Gu G., Lorrain D.S., Wei H., Cole R.L., Zhang X., Daggett L.P., Schaffhauser H.J., Bristow L.J., Lechner S.M. (2008). Distribution of metabotropic glutamate 2 and 3 receptors in the rat forebrain: implication in emotional responses and central disinhibition. Brain Res..

[bib15] Guillery R.W. (1995). Anatomical evidence concerning the role of the thalamus in corticocortical communication: a brief review. J. Anat..

[bib16] Harich S., Gross G., Bespalov A. (2007). Stimulation of the metabotropic glutamate 2/3 receptor attenuates social novelty discrimination deficits induced by neonatal phencyclidine treatment. Psychopharmacol. Berl..

[bib17] Harris R.M., Hendrickson A.E. (1987). Local circuit neurons in the rat ventrobasal thalamus–a GABA immunocytochemical study. Neuroscience.

[bib18] Harrison P.J., Lyon L., Sartorius L.J., Burnet P.W., Lane T.A. (2008). The group II metabotropic glutamate receptor 3 (mGluR3, mGlu3, GRM3): expression, function and involvement in schizophrenia. J. Psychopharmacol..

[bib19] Herman E.J., Bubser M., Conn P.J., Jones C.K. (2012). Metabotropic glutamate receptors for new treatments in schizophrenia. Handb. Exp. Pharmacol..

[bib20] Hermes M.L., Renaud L.P. (2010). Post- and presynaptic group II metabotropic glutamate receptors reduce neuronal excitability in rat midline paraventricular thalamic nucleus. J. Pharmacol. Exp. Ther..

[bib21] Holmes O., Houchin J. (1966). Units in the cerebral cortex of the anaesthetized rat and the correlations between their discharges. J. Physiol..

[bib22] Jahnsen H., Llinas R. (1984). Electrophysiological properties of guinea-pig thalamic neurones: an in vitro study. J. Physiol..

[bib23] Javitt D.C. (2009). Sensory processing in schizophrenia: neither simple nor intact. Schizophr. Bull..

[bib72] Johnson M.P., Baez M., Jagdmann G.E., Britton T.C., Large T.H., Callagaro D.O., Tizzano J.P., Monn J.A., Schoepp D.D. (2003). Discovery of allosteric potentiators for the metabotropic glutamate 2 receptor: synthesis and subtype selectivity of N-(4-(2-methoxyphenoxy)phenyl)-N-(2,2,2-trifluoroethylsylfonyl)pyrid-3-ylmethylamine. J. Med. Chem..

[bib24] Jones E.G. (2007). The Thalamus.

[bib25] Kay S.R., Fiszbein A., Opler L.A. (1987). The positive and negative syndrome scale (PANSS) for schizophrenia. Schizophr. Bull..

[bib26] Kingston A.E., Ornstein P.L., Wright R.A., Johnson B.G., Mayne N.G., Burnett J.P., Belagaje R., Wu S., Schoepp D.D. (1998). LY341495 is a nanomolar potent and selective antagonist of group II metabotropic glutamate receptors. Neuropharmacology.

[bib27] Kinon B.J., Zhang L., Millen B.A., Osuntokun O.O., Williams J.E., Kollack-Walker S., Jackson K., Kryzhanovskaya L., Jarkova N. (2011). A multicenter, inpatient, phase 2, double-blind, placebo-controlled dose-ranging study of LY2140023 monohydrate in patients with DSM-IV schizophrenia. J. Clin. Psychopharmacol..

[bib28] Kuroda M., Yokofujita J., Murakami K. (1998). An ultrastructural study of the neural circuit between the prefrontal cortex and the mediodorsal nucleus of the thalamus. Prog. Neurobiol..

[bib29] Lewis D.A., Lieberman J.A. (2000). Catching up on schizophrenia: natural history and neurobiology. Neuron.

[bib30] Llinas R., Jahnsen H. (1982). Electrophysiology of mammalian thalamic neurones in vitro. Nature.

[bib31] Lu S.M., Guido W., Sherman S.M. (1992). Effects of membrane voltage on receptive field properties of lateral geniculate neurons in the cat: contributions of the low-threshold Ca2+ conductance. J. Neurophysiol..

[bib32] Marenco J.P. (2012). Failure to deliver ICD shocks after a failed discharge despite redetection of rapid ventricular tachycardia? what is the cause?. Pacing Clin. Electrophysiol..

[bib33] Minzenberg M.J., Laird A.R., Thelen S., Carter C.S., Glahn D.C. (2009). Meta-analysis of 41 functional neuroimaging studies of executive function in schizophrenia. Arch. Gen. Psychiatry.

[bib34] Mitelman S.A., Byne W., Kemether E.M., Hazlett E.A., Buchsbaum M.S. (2005). Metabolic disconnection between the mediodorsal nucleus of the thalamus and cortical Brodmann's areas of the left hemisphere in schizophrenia. Am. J. Psychiatry.

[bib35] Mitelman S.A., Byne W., Kemether E.M., Newmark R.E., Hazlett E.A., Haznedar M.M., Buchsbaum M.S. (2006). Metabolic thalamocortical correlations during a verbal learning task and their comparison with correlations among regional volumes. Brain Res..

[bib36] Moghaddam B., Adams B.W. (1998). Reversal of phencyclidine effects by a group II metabotropic glutamate receptor agonist in rats. Science.

[bib37] Moldrich R.X., Chapman A.G., De Sarro G., Meldrum B.S. (2003). Glutamate metabotropic receptors as targets for drug therapy in epilepsy. Eur. J. Pharmacol..

[bib38] Mounce J., Luo L., Caprihan A., Liu J., Perrone-Bizzozero N.I., Calhoun V.D. (2014). Association of GRM3 polymorphism with white matter integrity in schizophrenia. Schizophr. Res..

[bib39] Nikiforuk A., Popik P., Drescher K.U., van Gaalen M.M., Relo A.L., Mezler M., Marek G., Schoemaker H., Gross G., Bespalov A. (2010). Effects of a positive allosteric modulator of mGlu2 receptors LY487379 on cognitive flexibility and impulsive-like responding in rats. J. Pharmacol. Exp. Ther..

[bib40] Nordquist R.E., Steckler T., Wettstein J.G., Mackie C., Spooren W. (2008). Metabotropic glutamate receptor modulation, translational methods, and biomarkers: relationships with anxiety. Psychopharmacol. Berl..

[bib41] Ohara P.T., Lieberman A.R. (1993). Some aspects of the synaptic circuitry underlying inhibition in the ventrobasal thalamus. J. Neurocytol..

[bib42] Parnaudeau S., O'Neill P.K., Bolkan S.S., Ward R.D., Abbas A.I., Roth B.L., Balsam P.D., Gordon J.A., Kellendonk C. (2013). Inhibition of mediodorsal thalamus disrupts thalamofrontal connectivity and cognition. Neuron.

[bib43] Patil S.T., Zhang L., Martenyi F., Lowe S.L., Jackson K.A., Andreev B.V., Avedisova A.S., Bardenstein L.M., Gurovich I.Y., Morozova M.A., Mosolov S.N., Naznanov N.G., Reznik A.M., Smulevich A.B., Tochilov V.A., Johnsonm B.G., Monn J.A., Schoepp D.D. (2007). Activation of mGlu2/3 receptors as a new approach to treat schizophrenia: a randomized phase 2 clinical trial. Nat. Med..

[bib44] Paxinos G., Watson C. (1998). The Rat Brain in Stereotaxic Co-ordinates.

[bib45] Petralia R.S., Wang Y.X., Niedzielski A.S., Wenthold R.J. (1996). The metabotropic glutamate receptors, mGluR2 and mGluR3, show unique postsynaptic, presynaptic and glial localizations. Neuroscience.

[bib46] Poisik O., Raju D.V., Verreault M., Rodriguez A., Abeniyi O.A., Conn P.J., Smith Y. (2005). Metabotropic glutamate receptor 2 modulates excitatory synaptic transmission in the rat globus pallidus. Neuropharmacology.

[bib47] Ralston H.H.I. (1983). The synaptic organization of the ventrobasal thalamus in the rat, cat and monkey. Somatosensory Integration in the Thalamus.

[bib48] Ramcharan E.J., Gnadt J.W., Sherman S.M. (2000). Burst and tonic firing in thalamic cells of unanesthetized, behaving monkeys. Vis. Neurosci..

[bib49] Rivadulla C., Martinez L., Grieve K.L., Cudeiro J. (2003). Receptive field structure of burst and tonic firing in feline lateral geniculate nucleus. J. Physiol..

[bib50] Saalmann Y.B., Kastner S. (2011). Cognitive and perceptual functions of the visual thalamus. Neuron.

[bib51] Saalmann Y.B., Pinsk M.A., Wang L., Li X., Kastner S. (2012). The pulvinar regulates information transmission between cortical areas based on attention demands. Science.

[bib52] Salt T.E. (1989). Gamma-aminobutyric acid and afferent inhibition in the cat and rat ventrobasal thalamus. Neuroscience.

[bib53] Salt T.E., Eaton S.A. (1995). Distinct presynaptic metabotropic receptors for L-AP4 and CCG1 on GABAergic terminals: pharmacological evidence using novel alpha-methyl derivative mGluR antagonists, MAP4 and MCCG, in the rat thalamus in vivo. Neuroscience.

[bib54] Salt T.E., Eaton S.A. (1995). Modulation of sensory neurone excitatory and inhibitory responses in the ventrobasal thalamus by activation of metabotropic excitatory amino acid receptors. Neuropharmacology.

[bib55] Salt T.E., Eaton S.A., Turner J.P. (1996). Characterization of the metabotropic glutamate receptors (mGluRs) which modulate GABA-mediated inhibition in the ventrobasal thalamus. Neurochem.. Int..

[bib56] Salt T.E., Turner J.P. (1998). Modulation of sensory inhibition in the ventrobasal thalamus via activation of group II metabotropic glutamate receptors by 2R,4R-aminopyrrolidine-2,4-dicarboxylate. Exp. Brain Res..

[bib57] Schaffhauser H., Rowe B.A., Morales S., Chavez-Noriega L.E., Yin R., Jachec C., Rao S.P., Bain G., Pinkerton A.B., Vernier J.M., Bristow L.J., Varney M.A., Daggett L.P. (2003). Pharmacological characterization and identification of amino acids involved in the positive modulation of metabotropic glutamate receptor subtype 2. Mol. Pharmacol..

[bib58] Schoepp D.D., Jane D.E., Monn J.A. (1999). Pharmacological agents acting at subtypes of metabotropic glutamate receptors. Neuropharmacology.

[bib59] Schoepp D.D., Wright R.A., Levine L.R., Gaydos B., Potter W.Z. (2003). LY354740, an mGlu2/3 receptor agonist as a novel approach to treat anxiety/stress. Stress.

[bib60] Sherman S.M., Guillery R.W. (2001). Exploring the Thalamus.

[bib61] Sidorov B.M., Podachin V.P. (1982). Character of evoked responses of the dorsomedial thalamic nucleus to stimulation of the periamygdalar cortex and area amygdalar anterior in rats. Neirofiziologiya.

[bib62] Simon A.P., Poindessous-Jazat F., Dutar P., Epelbaum J., Bassant M.H. (2006). Firing properties of anatomically identified neurons in the medial septum of anesthetized and unanesthetized restrained rats. J. Neurosci..

[bib63] Stauffer V.L., Millen B.A., Andersen S., Kinon B.J., Lagrandeur L., Lindenmayer J.P., Gomez J.C. (2013). Pomaglumetad methionil: no significant difference as an adjunctive treatment for patients with prominent negative symptoms of schizophrenia compared to placebo. Schizophr. Res..

[bib64] Turner J.P., Salt T.E. (2003). Group II and III metabotropic glutamate receptors and the control of the nucleus reticularis thalami input to rat thalamocortical neurones in vitro. Neuroscience.

[bib65] Uhlhaas P.J., Roux F., Singer W. (2013). Thalamocortical synchronization and cognition: implications for schizophrenia?. Neuron.

[bib66] Varga C., Sik A., Lavallee P., Deschenes M. (2002). Dendroarchitecture of relay cells in thalamic barreloids: a substrate for cross-whisker modulation. J. Neurosci..

[bib67] Wang W., Jones H.E., Andolina I.M., Salt T.E., Sillito A.M. (2006). Functional alignment of feedback effects from visual cortex to thalamus. Nat. Neurosci..

[bib68] Watanabe Y., Funahashi S. (2012). Thalamic mediodorsal nucleus and working memory. Neurosci. Biobehav. Rev..

[bib69] Woodward N.D., Karbasforoushan H., Heckers S. (2012). Thalamocortical dysconnectivity in schizophrenia. Am. J. Psychiatry.

[bib70] Wright R.A., Arnold M.B., Wheeler W.J., Ornstein P.L., Schoepp D.D. (2001). [3H]LY341495 binding to group II metabotropic glutamate receptors in rat brain. J. Pharmacol. Exp. Ther..

[bib71] Zipursky R.B. (2014). Why are outcomes in patients with schizophrenia so poor?. J. Clin. Psychiatry.

